# (*E*)-1-[(3-Bromo­phen­yl)imino­meth­yl]naphthalen-2-ol

**DOI:** 10.1107/S1600536812034824

**Published:** 2012-08-25

**Authors:** Tufan Akbal, Ayşen Ağar Alaman, Sümeyye Gümüş, Ahmet Erdönmez

**Affiliations:** a Ondokuz Mayıs University, Arts and Sciences Faculty, Department of Physics, 55139 Samsun, Turkey; b Ondokuz Mayıs University, Arts and Sciences Faculty, Department of Chemistry, 55139 Samsun, Turkey

## Abstract

The title compound, C_17_H_12_BrNO, exists in an enol–imine form and the mol­ecular structure features an intra­molecular O—H⋯N hydrogen bond. The dihedral angle between the benzene ring and the naphthalene ring system is 17.27 (15)°.

## Related literature

For general background to and applications of Schiff bases, see: Garnovski *et al.* (1993 ▶); Hamilton *et al.* (1987 ▶); Pyrz *et al.* (1985 ▶); Costamagna *et al.* (1992 ▶). For a related structure, see: Ünver *et al.* (2000 ▶).
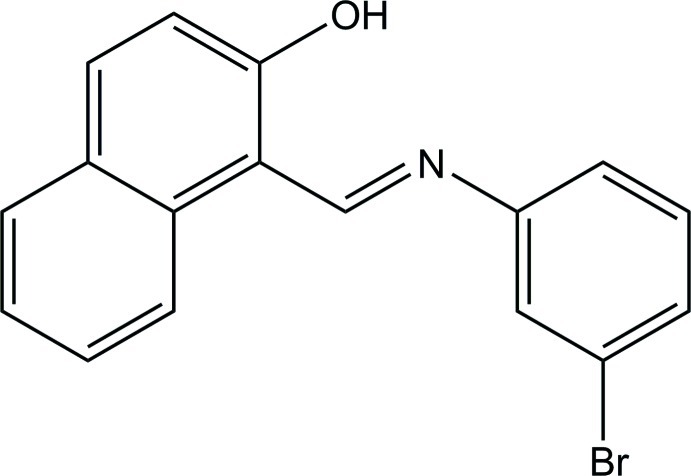


## Experimental

### 

#### Crystal data

C_17_H_12_BrNO
*M* *_r_* = 326.19
Monoclinic, 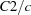

*a* = 31.3965 (19) Å
*b* = 4.8657 (2) Å
*c* = 19.0124 (11) Å
β = 107.772 (4)°
*V* = 2765.8 (3) Å^3^
*Z* = 8
Mo *K*α radiation
μ = 2.97 mm^−1^
*T* = 296 K
0.80 × 0.36 × 0.13 mm

#### Data collection

Stoe IPDS 2 diffractometer
Absorption correction: integration (*X-RED32*; Stoe & Cie, 2002 ▶) *T*_min_ = 0.421, *T*_max_ = 0.680
14469 measured reflections
2706 independent reflections
1992 reflections with *I* > 2σ(*I*)
*R*_int_ = 0.047

#### Refinement

*R*[*F* ^2^ > 2σ(*F* ^2^)] = 0.047
*wR*(*F* ^2^) = 0.117
*S* = 1.02
2706 reflections
181 parameters
H-atom parameters constrained
Δρ_max_ = 0.54 e Å^−3^
Δρ_min_ = −0.47 e Å^−3^

### 

Data collection: *X-AREA* (Stoe & Cie, 2002 ▶); cell refinement: *X-AREA*; data reduction: *X-RED32* (Stoe & Cie, 2002 ▶); program(s) used to solve structure: *WinGX* (Farrugia, 1999 ▶) and *SHELXS97* (Sheldrick, 2008 ▶); program(s) used to refine structure: *SHELXL97* (Sheldrick, 2008 ▶); molecular graphics: *ORTEP-3* for Windows (Farrugia, 1997 ▶); software used to prepare material for publication: *WinGX* and *PLATON* (Spek, 2009 ▶).

## Supplementary Material

Crystal structure: contains datablock(s) I, global. DOI: 10.1107/S1600536812034824/is5182sup1.cif

Structure factors: contains datablock(s) I. DOI: 10.1107/S1600536812034824/is5182Isup2.hkl

Supplementary material file. DOI: 10.1107/S1600536812034824/is5182Isup3.mol

Supplementary material file. DOI: 10.1107/S1600536812034824/is5182Isup4.cml

Additional supplementary materials:  crystallographic information; 3D view; checkCIF report

## Figures and Tables

**Table 1:** Hydrogen-bond geometry (Å, °)

*D*—H⋯*A*	*D*—H	H⋯*A*	*D*⋯*A*	*D*—H⋯*A*
O1—H1*A*⋯N1	0.82	1.82	2.548 (4)	147

**Fig. 1.**
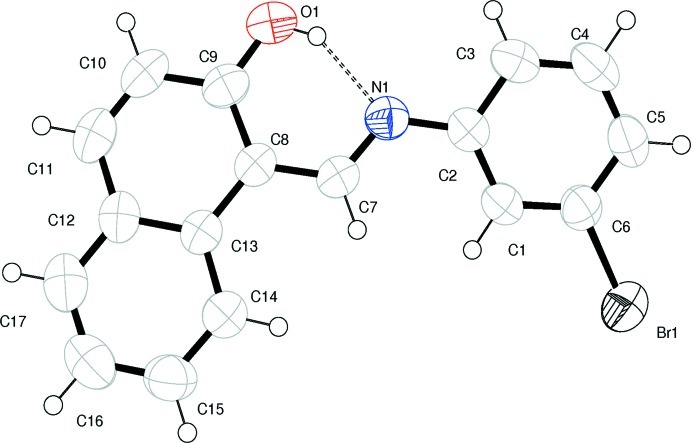
The title molecule with the atom-numbering scheme. The displacement ellipsoids are drawn at the 30% probability level.

**Fig. 2.**
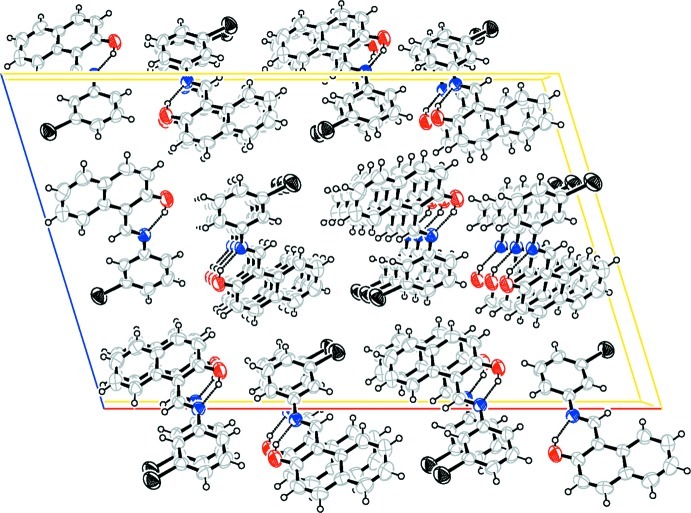
A view of the crystal packing of the title compound.

## supplementary crystallographic information

### Comment

Schiff bases from 2-hydroxy-1-naphthaldehyde have often been used as chelating ligands in the field coordination chemistry (Garnovski *et al.*, 1993). The Schiff base complexes have also been used in catalytic reactions (Hamilton *et al.*, 1987) and used as models for biological systems (Pyrz *et al.*, 1985; Costamagna *et al.*, 1992). There are two types of intramolecular hydrogen bonds in Schiff bases, namely keto-amine (N—H···O) and enol-imine (N···H—O) tautomeric forms.

The present X-ray investigation shows that the title compound, (I), prefers the enol-imine tautomeric form rather than the keto-amine tautomeric form (Fig. 1). The C9—O1 and C7—N1 bond lengths verify the enol-imine tautomeric form; these distances agree with the literature [1.310 (8) and 1.319 (6) Å; Ünver *et al.*, 2000], which also shows the enol-imine tautomeric form. The C6—Br1 bond length in (I) is also in a good agreement with the corresponding distance in the literature [1.904 (2) Å; Ünver *et al.*, 2000]. The molecule is non-planar. The dihedral angle between the two Schiff base moieties (C1–C6/N1) and (C7–C13/O1) is 16.27 (12)°. A view of the crystal packing of the title compound is shown in Fig. 2. π–π interactions between the centroids of the *Cg*1 and *Cg*2 rings [distance between ring centroids = 4.6002 (19) Å], and the *Cg*2 and *Cg*3 rings [distance between ring centroids = 4.805 (2) Å], stack the molecules along the *b*-axis. *Cg*1, *Cg*2 and *Cg*3 are the centroids of the C1–C6, C8–C13 and C12–C17 rings, respectively.

### Experimental

The compound (*E*)-1-[(3-bromophenyllimino)methyl]naphthalen-2-ol was prepared by refluxing a mixture of a solution containing 2-hydroxy-1-naphthaldehyde (17.2 mg, 0.100 mmol) in 30 ml absolute ethanol and a solution containing 3-bromoaniline (17.2 mg, 0.100 mmol) in 20 ml absolute ethanol. The reaction mixture was stirred for 4 h under reflux. Single crystals of the title compound for X-ray analysis were obtained by slow evaporation of an ethanol solution (yield 72%; m.p. 398–400 K).

### Refinement

H atoms were located in a difference Fourier map and then were treated using riding models, with C—H = 0.93 Å and O—H = 0.82 Å, and with *U*_iso_(H) = 1.2*U*_eq_(C or O).

### Crystal data

**Table:**

C_17_H_12_BrNO	*F*(000) = 1312
*M**_r_* = 326.19	*D*_x_ = 1.567 Mg m^−^^3^
Monoclinic, *C*2/*c*	Melting point = 398–400 K
Hall symbol: -C 2yc	Mo *K*α radiation, λ = 0.71073 Å
*a* = 31.3965 (19) Å	Cell parameters from 14469 reflections
*b* = 4.8657 (2) Å	θ = 1.4–26°
*c* = 19.0124 (11) Å	µ = 2.97 mm^−^^1^
β = 107.772 (4)°	*T* = 296 K
*V* = 2765.8 (3) Å^3^	Needle, yellow
*Z* = 8	0.80 × 0.36 × 0.13 mm

### Data collection

**Table:**

Stoe IPDS 2 diffractometer	2706 independent reflections
Radiation source: fine-focus sealed tube	1992 reflections with *I* > 2σ(*I*)
Graphite monochromator	*R*_int_ = 0.047
ω scans	θ_max_ = 26.0°, θ_min_ = 1.4°
Absorption correction: integration (*X-RED32*; Stoe & Cie, 2002)	*h* = −38→38
*T*_min_ = 0.421, *T*_max_ = 0.680	*k* = −6→5
14469 measured reflections	*l* = −23→23

### Refinement

**Table:**

Refinement on *F*^2^	Secondary atom site location: difference Fourier map
Least-squares matrix: full	Hydrogen site location: inferred from neighbouring sites
*R*[*F*^2^ > 2σ(*F*^2^)] = 0.047	H-atom parameters constrained
*wR*(*F*^2^) = 0.117	*w* = 1/[σ^2^(*F*_o_^2^) + (0.0492*P*)^2^ + 3.7545*P*] where *P* = (*F*_o_^2^ + 2*F*_c_^2^)/3
*S* = 1.02	(Δ/σ)_max_ = 0.001
2706 reflections	Δρ_max_ = 0.54 e Å^−^^3^
181 parameters	Δρ_min_ = −0.47 e Å^−^^3^
0 restraints	Extinction correction: *SHELXL97* (Sheldrick, 2008)
Primary atom site location: structure-invariant direct methods	Extinction coefficient: 0

### Special details

**Table:**

Geometry. All e.s.d.'s (except the e.s.d. in the dihedral angle between two l.s. planes) are estimated using the full covariance matrix. The cell e.s.d.'s are taken into account individually in the estimation of e.s.d.'s in distances, angles and torsion angles; correlations between e.s.d.'s in cell parameters are only used when they are defined by crystal symmetry. An approximate (isotropic) treatment of cell e.s.d.'s is used for estimating e.s.d.'s involving l.s. planes.
Refinement. Refinement of *F*^2^ against ALL reflections. The weighted *R*-factor *wR* and goodness of fit *S* are based on *F*^2^, conventional *R*-factors *R* are based on *F*, with *F* set to zero for negative *F*^2^. The threshold expression of *F*^2^ > σ(*F*^2^) is used only for calculating *R*-factors(gt) *etc*. and is not relevant to the choice of reflections for refinement. *R*-factors based on *F*^2^ are statistically about twice as large as those based on *F*, and *R*- factors based on ALL data will be even larger.

### Fractional atomic coordinates and isotropic or equivalent isotropic displacement parameters (Å^2^)

**Table:**

	*x*	*y*	*z*	*U*_iso_*/*U*_eq_	
Br1	0.051469 (14)	0.50605 (11)	0.33169 (2)	0.0901 (2)	
N1	0.16786 (9)	−0.1439 (6)	0.51791 (14)	0.0553 (7)	
O1	0.22611 (8)	−0.3797 (6)	0.62422 (15)	0.0750 (7)	
H1A	0.2164	−0.2890	0.5863	0.112*	
C1	0.11528 (11)	0.1582 (7)	0.42764 (17)	0.0533 (8)	
H1	0.0910	0.0929	0.4410	0.064*	
C2	0.15784 (11)	0.0544 (6)	0.46103 (16)	0.0504 (8)	
C3	0.19318 (12)	0.1518 (9)	0.4388 (2)	0.0642 (9)	
H3	0.2217	0.0813	0.4602	0.077*	
C4	0.18636 (14)	0.3517 (9)	0.3854 (2)	0.0721 (11)	
H4	0.2104	0.4153	0.3712	0.087*	
C5	0.14434 (14)	0.4590 (8)	0.35266 (19)	0.0664 (10)	
H5	0.1397	0.5952	0.3168	0.080*	
C6	0.10943 (12)	0.3588 (8)	0.37456 (17)	0.0573 (8)	
C7	0.13773 (11)	−0.2878 (7)	0.53452 (16)	0.0509 (7)	
H7	0.1079	−0.2624	0.5071	0.061*	
C9	0.19303 (11)	−0.5211 (7)	0.63643 (18)	0.0583 (8)	
C8	0.14817 (10)	−0.4839 (6)	0.59306 (16)	0.0493 (7)	
C10	0.20366 (13)	−0.7094 (8)	0.69535 (19)	0.0672 (10)	
H10	0.2332	−0.7301	0.7245	0.081*	
C11	0.17155 (14)	−0.8593 (9)	0.70984 (19)	0.0671 (10)	
H11	0.1796	−0.9824	0.7491	0.080*	
C12	0.12580 (12)	−0.8380 (7)	0.66784 (17)	0.0569 (8)	
C13	0.11370 (11)	−0.6490 (7)	0.60843 (16)	0.0495 (7)	
C14	0.06822 (12)	−0.6362 (9)	0.5669 (2)	0.0658 (9)	
H14	0.0592	−0.5161	0.5271	0.079*	
C15	0.03683 (14)	−0.7972 (9)	0.5839 (2)	0.0783 (11)	
H15	0.0070	−0.7845	0.5556	0.094*	
C16	0.04908 (16)	−0.9769 (9)	0.6424 (3)	0.0837 (12)	
H16	0.0275	−1.0840	0.6537	0.100*	
C17	0.09253 (16)	−0.9979 (8)	0.6835 (2)	0.0753 (11)	
H17	0.1005	−1.1207	0.7228	0.090*	

### Atomic displacement parameters (Å^2^)

**Table:**

	*U*^11^	*U*^22^	*U*^33^	*U*^12^	*U*^13^	*U*^23^
Br1	0.0739 (3)	0.0978 (4)	0.0872 (3)	0.0032 (3)	0.0079 (2)	0.0291 (3)
N1	0.0548 (16)	0.0576 (17)	0.0552 (15)	0.0016 (14)	0.0192 (12)	−0.0048 (13)
O1	0.0496 (14)	0.0885 (19)	0.0819 (17)	0.0024 (14)	0.0128 (12)	0.0074 (15)
C1	0.0540 (18)	0.056 (2)	0.0533 (17)	−0.0079 (16)	0.0218 (14)	−0.0053 (15)
C2	0.0569 (18)	0.0463 (19)	0.0498 (16)	−0.0055 (15)	0.0191 (14)	−0.0095 (13)
C3	0.055 (2)	0.070 (2)	0.070 (2)	−0.0105 (18)	0.0237 (17)	−0.0091 (19)
C4	0.073 (3)	0.082 (3)	0.072 (2)	−0.024 (2)	0.038 (2)	−0.008 (2)
C5	0.083 (3)	0.066 (2)	0.0549 (18)	−0.017 (2)	0.0270 (18)	0.0001 (17)
C6	0.062 (2)	0.062 (2)	0.0465 (16)	−0.0071 (18)	0.0135 (15)	−0.0046 (15)
C7	0.0500 (17)	0.0521 (19)	0.0489 (16)	0.0043 (15)	0.0126 (14)	−0.0053 (14)
C8	0.0534 (17)	0.0482 (17)	0.0456 (15)	0.0062 (16)	0.0138 (13)	−0.0061 (14)
C9	0.0557 (19)	0.059 (2)	0.0581 (18)	0.0081 (18)	0.0138 (15)	−0.0089 (16)
C10	0.063 (2)	0.073 (3)	0.0559 (19)	0.018 (2)	0.0042 (17)	0.0007 (18)
C11	0.084 (3)	0.063 (2)	0.0507 (18)	0.017 (2)	0.0150 (18)	0.0037 (17)
C12	0.076 (2)	0.0477 (19)	0.0511 (17)	0.0081 (18)	0.0257 (16)	−0.0027 (15)
C13	0.0557 (18)	0.0456 (17)	0.0483 (16)	0.0065 (15)	0.0175 (14)	−0.0060 (14)
C14	0.061 (2)	0.066 (2)	0.069 (2)	0.003 (2)	0.0183 (17)	0.0064 (18)
C15	0.058 (2)	0.081 (3)	0.096 (3)	−0.001 (2)	0.025 (2)	0.002 (2)
C16	0.087 (3)	0.077 (3)	0.099 (3)	−0.012 (3)	0.046 (3)	0.001 (3)
C17	0.096 (3)	0.064 (2)	0.073 (2)	0.007 (3)	0.036 (2)	0.009 (2)

### Geometric parameters (Å, º)

**Table:**

Br1—C6	1.893 (4)	C9—C10	1.406 (5)
N1—C7	1.291 (4)	C9—C8	1.410 (4)
N1—C2	1.411 (4)	C10—H10	0.9300
O1—C9	1.324 (4)	C11—C10	1.339 (6)
O1—H1A	0.8200	C11—C12	1.418 (5)
C1—C6	1.375 (5)	C11—H11	0.9300
C1—C2	1.388 (4)	C12—C17	1.405 (5)
C1—H1	0.9300	C12—C13	1.416 (4)
C3—C4	1.375 (6)	C13—C14	1.406 (5)
C3—C2	1.386 (5)	C13—C8	1.447 (5)
C3—H3	0.9300	C14—C15	1.372 (6)
C4—H4	0.9300	C14—H14	0.9300
C5—C4	1.378 (6)	C15—H15	0.9300
C5—H5	0.9300	C16—C17	1.353 (6)
C6—C5	1.375 (5)	C16—C15	1.373 (6)
C7—C8	1.426 (4)	C16—H16	0.9300
C7—H7	0.9300	C17—H17	0.9300
			
C7—N1—C2	123.3 (3)	O1—C9—C8	121.9 (3)
C9—O1—H1A	109.5	C10—C9—C8	120.1 (3)
C6—C1—C2	119.2 (3)	C11—C10—C9	120.5 (3)
C6—C1—H1	120.4	C11—C10—H10	119.7
C2—C1—H1	120.4	C9—C10—H10	119.7
C3—C2—C1	119.0 (3)	C10—C11—C12	122.8 (3)
C3—C2—N1	117.1 (3)	C10—C11—H11	118.6
C1—C2—N1	123.9 (3)	C12—C11—H11	118.6
C4—C3—C2	120.6 (4)	C17—C12—C13	119.5 (3)
C4—C3—H3	119.7	C17—C12—C11	122.1 (3)
C2—C3—H3	119.7	C13—C12—C11	118.4 (3)
C3—C4—C5	120.9 (4)	C14—C13—C12	117.1 (3)
C3—C4—H4	119.6	C14—C13—C8	123.8 (3)
C5—C4—H4	119.6	C12—C13—C8	119.1 (3)
C6—C5—C4	118.1 (4)	C15—C14—C13	121.5 (4)
C6—C5—H5	121.0	C15—C14—H14	119.3
C4—C5—H5	121.0	C13—C14—H14	119.3
C1—C6—C5	122.3 (3)	C14—C15—C16	120.7 (4)
C1—C6—Br1	118.7 (3)	C14—C15—H15	119.7
C5—C6—Br1	119.1 (3)	C16—C15—H15	119.7
N1—C7—C8	122.9 (3)	C17—C16—C15	119.9 (4)
N1—C7—H7	118.6	C17—C16—H16	120.0
C8—C7—H7	118.6	C15—C16—H16	120.0
C9—C8—C7	119.5 (3)	C16—C17—C12	121.3 (4)
C9—C8—C13	119.1 (3)	C16—C17—H17	119.3
C7—C8—C13	121.4 (3)	C12—C17—H17	119.3
O1—C9—C10	118.0 (3)		
			
C2—N1—C7—C8	−178.8 (3)	C4—C3—C2—N1	−177.9 (3)
C10—C11—C12—C17	179.6 (4)	C6—C1—C2—C3	−1.3 (5)
C10—C11—C12—C13	−0.4 (5)	C6—C1—C2—N1	177.6 (3)
C17—C12—C13—C14	−1.1 (5)	C7—N1—C2—C3	−166.9 (3)
C11—C12—C13—C14	178.8 (3)	C7—N1—C2—C1	14.2 (5)
C17—C12—C13—C8	179.6 (3)	C1—C6—C5—C4	0.3 (5)
C11—C12—C13—C8	−0.4 (4)	Br1—C6—C5—C4	179.0 (3)
O1—C9—C8—C7	−0.4 (5)	C12—C11—C10—C9	−0.1 (6)
C10—C9—C8—C7	178.6 (3)	O1—C9—C10—C11	−179.5 (3)
O1—C9—C8—C13	178.8 (3)	C8—C9—C10—C11	1.5 (5)
C10—C9—C8—C13	−2.2 (5)	C12—C13—C14—C15	0.9 (5)
N1—C7—C8—C9	1.5 (5)	C8—C13—C14—C15	−179.9 (3)
N1—C7—C8—C13	−177.7 (3)	C2—C3—C4—C5	−0.2 (6)
C14—C13—C8—C9	−177.5 (3)	C6—C5—C4—C3	−0.5 (6)
C12—C13—C8—C9	1.7 (4)	C13—C14—C15—C16	−0.1 (6)
C14—C13—C8—C7	1.7 (5)	C17—C16—C15—C14	−0.5 (7)
C12—C13—C8—C7	−179.1 (3)	C15—C16—C17—C12	0.3 (6)
C2—C1—C6—C5	0.7 (5)	C13—C12—C17—C16	0.6 (6)
C2—C1—C6—Br1	−178.1 (2)	C11—C12—C17—C16	−179.4 (4)
C4—C3—C2—C1	1.1 (5)		

### Hydrogen-bond geometry (Å, º)

**Table:**

*D*—H···*A*	*D*—H	H···*A*	*D*···*A*	*D*—H···*A*
O1—H1*A*···N1	0.82	1.82	2.548 (4)	147
